# The Australian longitudinal study on male health-methods

**DOI:** 10.1186/s12889-016-3698-1

**Published:** 2016-10-31

**Authors:** Dianne Currier, Jane Pirkis, John Carlin, Louisa Degenhardt, Shyamali C. Dharmage, Billie Giles-Corti, Ian Gordon, Lyle Gurrin, Jane Hocking, Anne Kavanagh, Louise A. Keogh, Rachel Koelmeyer, Anthony D. LaMontagne, Marisa Schlichthorst, George Patton, Lena Sanci, Matthew J. Spittal, David M. Studdert, Joanne Williams, Dallas R. English

**Affiliations:** 1 Centre for Epidemiology and Biostatistics, Melbourne School of Population and Global Health, The University of Melbourne, Melbourne, 3010 Australia; 2 Centre for Mental Health, Melbourne School of Population and Global Health, The University of Melbourne, Melbourne, 3010 Australia; 3 National Drug and Alcohol Research Centre, University of New South Wales, Randwick, 2031 Australia; 4 Centre for Health Equity, Melbourne School of Population and Global Health, The University of Melbourne, Melbourne, 3010 Australia; 5 Statistical Consulting Centre, School of Mathematics and Statistics, The University of Melbourne, Melbourne, 3010 Australia; 6 School of Clinical Sciences, Monash University, Clayton, 3168 Australia; 7 School of Health & Social Development, Deakin University, Burwood, 3125 Australia; 8 Centre for Adolescent Health, Murdoch Childrens Research Institute, University of Melbourne, Melbourne, 3010 Australia; 9 Department of General Practice, Melbourne Medical School, The University of Melbourne, Melbourne, 3010 Australia; 10 Centre for Health Policy/PCOR, Stanford University School of Medicine, Stanford, 94305 USA; 11 Stanford Law School, Stanford, 94305 USA

## Abstract

**Background:**

The Australian Longitudinal Study on Male Health (Ten to Men) was established in 2011 to build the evidence base on male health to inform policy and program development.

**Methods:**

Ten to Men is a national longitudinal study with a stratified multi-stage cluster random sample design and oversampling in rural and regional areas. Household recruitment was conducted from October 2013 to July 2014. Males who were aged 10 to 55 years residing in private dwellings were eligible to participate. Data were collected via self-completion paper questionnaires (participants aged 15 to 55) and by computer-assisted personal interview (boys aged 10 to 14). Household and proxy health data for boys were collected from a parent via a self-completion paper-based questionnaire. Questions covered socio-demographics, health status, mental health and wellbeing, health behaviours, social determinants, and health knowledge and service use.

**Results:**

A cohort of 15,988 males aged between 10 and 55 years was recruited representing a response fraction of 35 %.

**Conclusion:**

Ten to Men is a unique resource for investigating male health and wellbeing. Wave 1 data are available for approved research projects.

## Background

The disparity in premature mortality and preventable disease burden between males and females, and between different groups of males, is well-documented in Australia and internationally [1–6]. Life-limiting diseases, particularly many cancers and cardio-vascular disease, suicide, and unintentional injuries are more prevalent in males than females, as are known health risk factors including obesity, tobacco and alcohol use [1, 5, 6]. The 2010 Australian National Male Health Policy identified a need to strengthen the evidence base on male health in order to inform the development of programs and policies targeting such disparities [7].

Australia is well served with respect to population health research, however there are notable gaps with respect to male health. The major Australia-wide population health studies are repeat cross-sectional surveys [8–11]. While large in scale, being cross-sectional these studies are unable to investigate causal pathways and observe developmental trajectories across the life-course, both of which are best addressed by longitudinal data. Current national longitudinal studies in Australia do not cover male health in sufficient depth or breadth as they are restricted by age group [12–14], gender [15], have limited coverage of health and wellbeing [16, 17], or focus on a narrowly defined set of health conditions or behaviours [18, 19]. Studies focussing exclusively on males in Australia have been few, small scale and often restricted to male-specific physical conditions [20–24].

Internationally, research on male health follows a similar pattern, notwithstanding two large-scale and long-running cohort studies of doctors and medical professionals in the US [25, 26], and more recent all-male cohort studies in the Asia region [27, 28]. However, these studies are not national population studies and all are restricted to adult males. Moreover social and cultural contexts are very different between Australia and these countries, as are health systems, which limits the extent to which important research questions regarding social determinants and service use in the Australia context can be addressed by those data.

Responding to the National Male Health Policy, in 2011 the Australian Government Department of Health provided funding to establish the Australian Longitudinal Study on Male Health (Ten to Men) a national longitudinal study of a large cohort of males aged 10 to 55 years. Ten to Men has been designed to provide a resource for investigating the complex interactions of social, environmental, and individual factors over the life course to elucidate the causal pathways leading to premature mortality and greater preventable disease morbidity in males. This paper describes the methodological details including sampling, recruitment and data collection of the baseline wave of Ten to Men.

## Objectives

The principal objectives of Ten to Men align closely with those stated in Australia’s 2010 National Male Health Policy [7]. They are to:

- examine male health and its key determinants including social, economic, environmental and behavioural factors that affect the length and quality of life of Australian males;
- address a range of key research questions about the health of Australian males, including their health behaviours, risk and protective factors, key life transition points, social and economic environments in which males work and live, and use of health and other services; and
- identify policy opportunities for improving the health and wellbeing of males and providing support for males at key life stages, particularly those at risk of poor health.

## Methods

### Design

Ten to Men is a national longitudinal cohort study of males aged 10–55 years at recruitment. Recruitment and Wave 1 data collection took place from October 2013 to July 2014. Participants were ascertained via household recruitment. Wave 1 data were collected via a computer assisted personal interview (CAPI) for 10–14 year olds (“boys”) with a supplementary hard-copy questionnaire for a parent/guardian, and self-completion hardcopy questionnaires for 15–17 year olds (“young men”) and 18–55 year olds (“adults”). Linkage with administrative datasets is planned including Australia’s universal health and pharmaceutical insurance schemes. Wave 2 data collection is scheduled to commence in November 2015.

### Sampling

Australia is a large, but highly urbanised country. A simple random sample was ruled out as infeasible due to the prohibitive cost involved in conducting recruitment and interviewing activities in many widely-dispersed locations. Instead a clustered random sample design was chosen. Because it was not possible to include males from remote areas, it was decided to over-sample males from regional areas. This introduced the need to stratify by region. The sample therefore can be described as a stratified, multi-stage, cluster random sample with separate cluster samples drawn within each regional stratum.

Clusters were based on geographical units set out in the Australian Statistical Geographic Standards (ASGS) [29]. The main structure of the ASGS consists of five levels of hierarchical spatial units, all of which aggregate progressively upwards. Mesh Blocks are the smallest unit; they aggregate into Statistical Area 1 s (SA1s), which aggregate into SA2s, then SA3s, SA4s and finally the six States and two Territories. SA1s are the smallest unit at which data from the Australian Bureau of Statistics (ABS) 2011 Census of Population and Housing is available and have an average population of 400 persons (range 200–800). SA2s are medium-sized areas comprising groups of adjacent SA1s that represent a social/economic community unit with an average population of 10,000 persons (range 2,000-25,000) [29].

The ASGS also designates six remoteness areas (RAs): Major Cities, Inner Regional, Outer Regional, Remote, Very Remote and Migratory [30]. The RAs divide Australia into broad geographic regions on the basis of their relative access to services. Major Cities, Inner and Outer Regional RAs constituted the three regional strata in the sample, while Remote, Very Remote and Migratory RAs were excluded.

In the Major Cities stratum SA1s were the primary sampling unit and were sampled randomly with probability proportional to size (where “size” was the number of boys living in that SA1 according to the 2011 Census). It was assumed that the population size of the SA1s in the Major Cities stratum was not strongly associated with any particular covariate profiles that might lead to lack of representation of some of the covariate distributions. Across SA1s there was a positive correlation between the population sizes for boys and young men, and similarly for young men and adults, and for boys and adults. That is, SA1s with a larger number of boys were likely to have a larger number of young men and a larger number of adults.

Due to the prohibitive cost of face-to-face recruitment and interviewing in potentially widely dispersed regional SA1s, Inner and Outer Regional SA2s were the primary sampling unit and were randomly selected, once again with probability of selection proportional to size. This strategy was essentially the same as using the number of SA1s per SA2 as the measure of size since the correlation between the number of boys and the number of SA1s within an SA2 was 0.96. A fixed number of SA1s were then selected as a simple random sample within each of the selected SA2s. Sampling a fixed number of SA1s in each SA2 compensates for the higher initial probability of selection of a larger SA2 and delivers a sample in which each individual in the target population has an equal chance of selection. All eligible households within a sampled SA1 and all eligible males within a household were in-scope for inclusion.

Approximately 5.1 % of SA1s were removed from the final sampling frame. SA1s enumerated in the pilot studies were also excluded (1.4 %). A further 2,250 SA1s (4.1 %) were excluded because, as at the time of Wave 1 recruitment (October 2013), they formed part of three other national household studies and we sought to avoid recruitment difficulties and participant burden that may have arisen from overlap. As all three studies were probability samples it was considered that excluding overlapping SA1s would not introduce systematic bias (especially when viewed from a longitudinal, rather than cross-sectional perspective). With these exclusions the final sample frame comprised 50,236 SA1s.

Data were obtained from the Australian Bureau of Statistics on male population by study age groups, SA1 and SA2, and the three ASGS remoteness areas included. In order to oversample regional males 65 % of the sample was drawn from Major Cities, 20 % from Inner and 15 % from Outer Regional RAs (the population distribution being 70, 18 and 9 % respectively).

A total of 622 SA1s were enumerated. That number was determined by the available resources. The distribution of the 622 SA1s across regional strata was determined based on census data and response estimates from the pilot study and resulted in selection of 363 SA1s in Major Cities, 144 in Inner Regional RAs and 115 in Outer Regional RAs. The design of the sample did not aim to guarantee state/territory representation, but the final sample did include SA1s from every state and the two mainland territories.

### Recruitment

Fieldwork was undertaken by the research services organisation Roy Morgan Research. All eligible households in a sampled SA1 and all eligible males within a household were in-scope for inclusion. Table 1 gives inclusion and exclusion criteria for households and individuals.

**Table 1:** Inclusion and exclusion criteria for households and individuals

Households	
Inclusion	• All private dwellings in a selected SA1.
Exclusion	• Non-private dwellings or institutional settings (i.e., hotels, motels, hostels, hospitals, nursing homes, short-stay caravan parks, military bases/barracks, prisons, corrective facilities, boarding schools, university residences, and convents and monasteries.)
Individuals	
Inclusion	• Male (self-identified); • Aged 10–55 years at the time of recruitment; • Australian citizen or permanent resident; • Resident in a selected dwelling. NB: part-time residents in a selected dwelling (e.g., children in shared care arrangements) were eligible if they are resident at the time of recruitment of that household; • Sufficient proficiency in English to complete the study questionnaire/interview.
Exclusion	• Males usually residing in non-private dwellings or institutional settings • Non-Australian diplomats, non-Australian diplomatic staff and non-Australian members of their household; • Members of non-Australian defence forces stationed in Australia and their dependents; • Overseas visitors (persons who have stayed or intended to stay in Australia for less than one year).

SA1s were allocated to fieldworkers who attempted to make contact with every household in the SA1 by making up to three in-person visits on different days and times, including at least once on a weekend. On making contact the fieldworker ascertained if there were any resident in-scope males and, if so, attempted to recruit them into the study. For males aged 15 years and older who agreed to participate, or for whom another household member agreed on their behalf, a study pack was left including the hardcopy questionnaires, study information brochure, consent and contact forms and privacy envelopes. Fieldworkers made an appointment to return and retrieve the completed documents and where possible collected a contact phone number. Up to two attempts were made to retrieve documents after which reply-paid envelopes were left. A reminder phone call was made after leaving the reply-paid envelopes. For males aged 10–14, an appointment was made for an interviewer to return and conduct the CAPI. Up to three attempts were made to conduct the interview. While the interview was occurring the parent completed the Parent Questionnaire.

Fieldworkers collected additional information on households including the details of all in-scope males regardless of whether they were participating, relationships between males in the same household who agreed to participate, and refusal reasons for eligible but non-participating households.

Written informed consent was required from all participants and parents of participating males under 18 years, for survey completion. Consent for linkage to administrative datasets was also sought but was optional.

## Questionnaires

Five broad domains for the questionnaire content were identified: health status, mental health and wellbeing, health behaviours, social determinants of health, and health knowledge and service use. Table 2 provides an overview of the constructs included within each domain and for each age group. Where possible the same measure was used across all age groups.

**Table 2:** Key domains and constructs in the baseline instruments by age group

Domain	Construct	Adults (18–55)	Young Men (15–17)	Boys (10–14)	Parents of Boys
Health status	Lifetime diagnosis and 12 month prevalence of chronic conditions^a^	x	x		x
	Self-reported Health Status	x	x	x	x
	Height & Weight	x	x	x	
	Sleep		x	x	
	Injury	x	x		x
	Disability	x	x		x
	Sexual function	x			
	Prostate health	x			
	Pubertal Development		x	x	
	Acne		x	x	
Mental health & wellbeing	Lifetime diagnosis and 12 month prevalence of selected disorders	x	x		x
	Current Depression	x	x	x	
	Anxiety (GAD, Social Phobia)		x	x	
	Self-injury (suicidal)	x	x	x	
	Self-Injury (non-suicidal)		x	x	
	Life Satisfaction	x	x	x	
Health behaviours	Tobacco: lifetime & current use,	x	x	x	
	Alcohol: lifetime & current use dependence	x	x	x	
	Illicit drugs: lifetime & 12 month use	x	x	x	
	Supplements and over-the-counter medicines	x			
	Fruit & Vegetable intake	x	x	x	
	Physical Activity & Sedentary Behaviour	x	x	x	
	Sun exposure	x			
	Sexual Behaviour	x	x		
	Risk taking	x	x		x
	Intimate Partner Violence	x			
	Bullying		x	x	
Social determinants	Education	x	x	x	
	Parental Education		x	x	x
	Employment status	x	x		x
	Working conditions	x			
	Household income	x			x
	Financial pressures	x			x
	Housing type & tenure	x	x		x
	Household structure/shared cared arrangements	x	x	x	x
	Social support	x	x	x	
	Community engagement	x	x	x	x
	Life events	x	x		x
	Masculinity	x	x		
	Gender Role	x	x		
Health services & knowledge	Use	x	x		x
	Access	x			x
	Satisfaction	x			
	Health Information	x	x	x	

^a^ selected on the basis of disease burden, priority areas identified in National Male Health Policy, & emerging areas of research interest

Where available, validated scales or questions were used or items were drawn from other large health studies. If no suitable measures were available the study investigators developed the questions. All such novel questions were subjected to cognitive testing and had their performance evaluated in pilot testing.

### Ethics approval

The Human Research Ethics Committee at the University of Melbourne approved the study. Ethics approval was obtained from the Australian Government Department of Health to link data from the Medicare Benefits Schedule and the Pharmaceutical Benefits Schedule.

### Pilot studies

Two pilot studies were conducted to determine the optimal recruitment method, test questionnaire performance and trial operational protocols. Roy Morgan Research conducted the fieldwork for both pilot studies.

Pilot Study 1 (Oct 2012) tested a mail-out method with postcodes as the principal sampling unit. Six postcodes in South Australia and Victoria were included. Potential participants were identified from the national health insurance (Medicare) database. An invitation to participate along with the hard-copy questionnaires and reply-paid envelopes was mailed to 1250 adults and 500 young men. Parents of 500 boys were mailed an invitation to contact the study to arrange for an interviewer to travel to their home and conduct the interview with the boy. The use of pre-notification letters and reminders was trialled. Response was well below expectations at 6.3 % (adults 5.8 %; young men 5.4 %; boys 7.7 %) and no pre-notification/reminder combination trialled showed any significant advantage in terms of increased response.

Pilot Study 2 (Feb 2013) trialled the household recruitment method, with SA1s as the primary sampling unit. Four SA1s in South Australia and Victoria were included. As with the main data collection protocol, fieldworkers made up to three visits to contact households, and having made contact and established the household was in-scope provided the age-relevant questionnaire and supporting documents for males aged 15–55. They then made up to two attempts to retrieve the completed questionnaires. For males aged 10–14, on making contact with a household the fieldworker made an appointment to conduct an interview. Up to three attempts were made to conduct the interview. During the interview a parent was provided the parent questionnaire to complete. 240 households were approached. A substantially higher response (42.3 %) was achieved (adults 43.9 %; young men 12.5 %; boys 50.0 %).

The small scale of Pilot Study 2 resulted in large confidence intervals around response estimates, particularly for boys and young men. Nevertheless, given the questions a 6.3 % response would inevitably raise around the external validity of the study it was decided to adopt the household recruitment method.

## Results

### Participants

#### Response

**Fig. 1:**
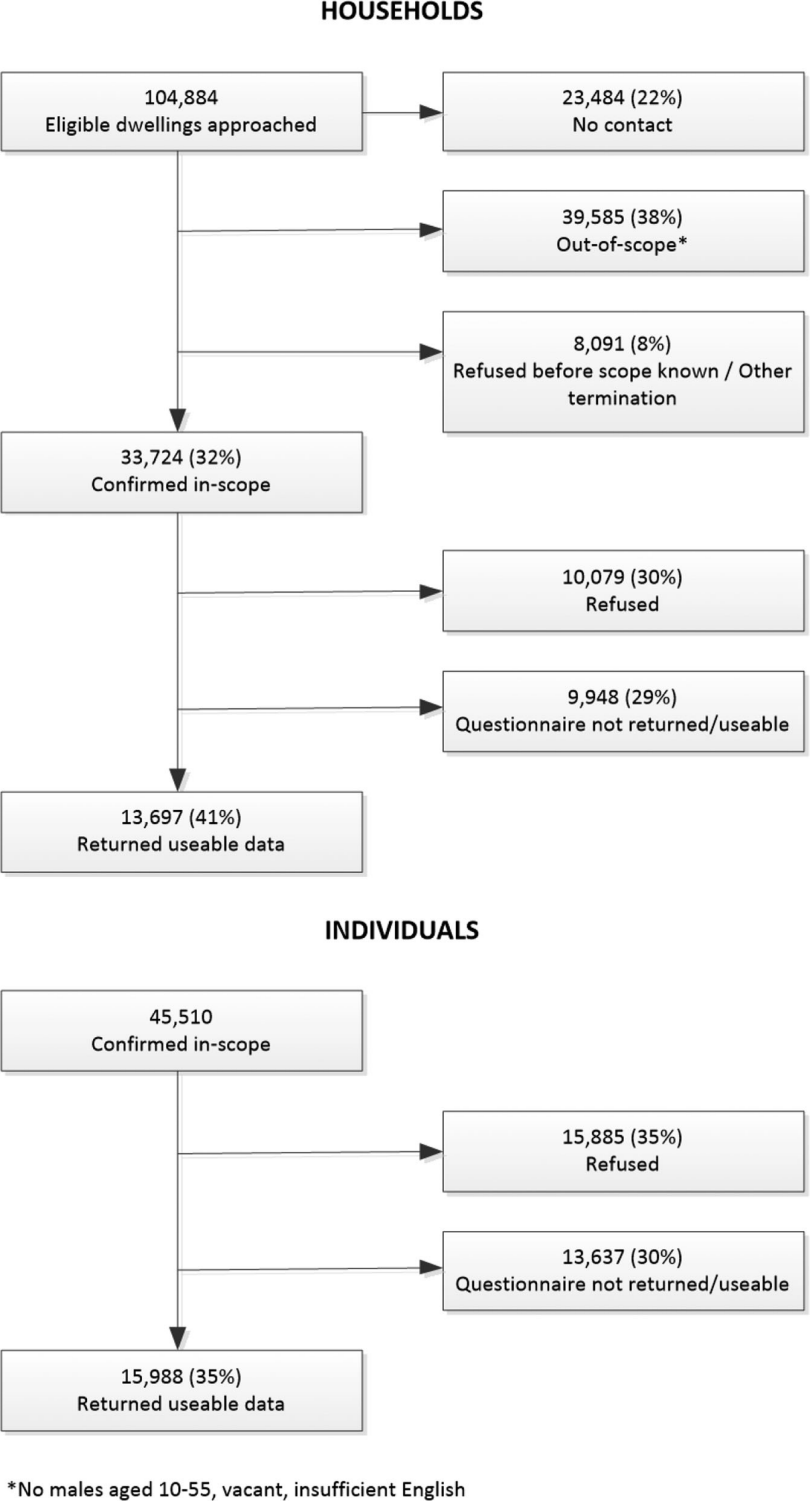
Household and Individual Response

Wave 1 fieldworkers approached 104,884 households. Contact was made with 81,400 (77.6 %) of which 33,724 (42 %) were confirmed to be in-scope. These in-scope households contained 45,510 in-scope males of whom 15,988 returned a useable questionnaire/ interview – a response fraction of 35 % of confirmed in-scope males. Figure 1 shows response for household and individual levels.

There was some variation in individual response by recruitment age-group and also by region – both overall and within each recruitment age-group (see Table 3).

**Table 3:** Response fractions by recruitment age group and regional strata

	Response %			
	Boys (10–14)	Young Men (15–17)	Adults (18–55)	All (10–55)
Major Cities	22 %	30 %	34 %	33 %
Inner Regional	29 %	35 %	41 %	40 %
Outer Regional	32 %	35 %	40 %	39 %
Total	25 %	32 %	36 %	35 %

Sample weights were calculated to address unequal sampling fractions and non-response. Full details on the development of the weights is available in Spittal et al. in this volume [31].

Refusal information was collected at the household level only. More than one refusal reason could be given. Among the 18,980 households refusing the main reasons recorded for refusal were ‘not interested/waste of time’ (18.8 %), ‘too busy’ (13.5 %) and ‘questions too personal’ (3.5 %).

#### Participant socio-demographic characteristics

Table 4 presents participant socio-demographic characteristics of the cohort (unweighted summary statistics) and comparisons with 2011 Census data. The Ten to Men cohort is older, more likely to be Australian-born, and more likely to live in regional areas reflecting the sample design. The proportion of indigenous Australians is similar to that recorded in the general population. A slightly larger proportion of the cohort lives in areas of highest disadvantage and slightly smaller proportion in areas of lowest disadvantage (1st and 5th socioeconomic quintiles respectively) [32].

**Table 4:** Comparison of Ten to Men participants with 2011 Census data for males age 10–55 in Major Cities, Inner Regional and Outer Regional RAs

Characteristic	Ten to men %^a^ *n* = 15,988	2011 census % *N* = 6,472,834
Age		
10–19	17.1	21.3
20–29	17.6	22.2
30–39	23.1	22.8
40–49	26.8	22.2
50–55	15.5	12.5
Australian born	77.7	69.2
Indigenous origin	2.8	2.2
Region		
Major City	57.6	74
Inner Regional	22.7	16.9
Outer Regional	19.6	9.1
Socio-economic Disadvantage (SEIFA^b^)		
1st Quintile	19.1	18.3
2nd Quintile	18.6	19.5
3rd Quintile	22.4	20.1
4th Quintile	20.3	20.5
5th Quintile	19.5	20.9

^a^ all proportions based on unweighted frequency counts

^b^ Australian Bureau of Statistics, Socio-Economic Indexes for Areas - Index of Relative Socio-Economic Disadvantage (IRSD). This is general socio-economic index produced from census data summarising range of indicators of economic and social disadvantage [32]. Lower IRSD scores indicate greater disadvantage

## Discussion

Ten to Men represents a major investment in building the knowledge base on male health to support the development of policies and programs addressing the premature mortality and preventable disease burden in Australian males. The breadth of data collected, particularly the focus on social determinants of health, the wide age range of the cohort and the oversampling of rural and regional males enables complex modelling of the relative importance of, and interactions between, health behaviours, environments and health outcomes over the life-course. In the short term, the cross-sectional Wave 1 data permit examination of associations between a wide variety of individual and social factors and health status indicators; this information will be valuable in generating hypotheses regarding causal pathways. In the longer term, as the longitudinal data accrue, these and other causal pathways can be investigated, shedding new light on the relationship between male health at various life stages and in a wide range of social, economic and geographic environments.

The overall response fraction of 35 %, while comparable to other more recently established health studies [33–35], limits the ability of Ten to Men to produce reliable prevalence estimates at a national level. However, response rate alone is not necessarily indicative of the reliability and quality of survey data [36] and reliable estimates can be achieved with appropriate weighting [37]. Moreover, the study was not designed as a national prevalence study, and there are other existing cross-sectional studies that can provide such information. Rather, Ten to Men’s strength will be as a platform for examining relationships between risk factors and health outcomes over time at the individual level. Lack of representativeness of a cohort is less important to the value of a longitudinal study than lack of heterogeneity within the cohort and selective attrition over time. If the baseline population is relatively homogeneous and determinants of interest have different effects in different population subgroups (including ones un- or under-represented in the cohort), the cohort may be ill-suited to supporting investigations of these relationships. Although there is evidence that the characteristics of Ten to Men participants do not perfectly match those of the general population of interest, there appears to be reasonable representation across key groups (at least those identifiable by observable characteristics). Nonetheless close attention must be paid to cohort heterogeneity as attrition occurs across successive waves.

### Limitations

The main limitation is the smaller number of males in the younger age bands, which will restrict the types of analysis possible and which may be exacerbated should attrition levels be high in subsequent waves. This will be particularly acute for low-prevalence disorders. No objective measures of health status or behaviours were possible in Wave 1 and, while widely used, issues of reliability have been raised with self-report health data across a range of outcomes and behaviours [38]. To address this Ten to Men questionnaires included, wherever possible, measures with demonstrated reliability and validity. Moreover, linkage to administrative data will provide objective data on frequency and type of service use. Males from culturally and linguistically diverse backgrounds were functionally excluded as study materials could not be produced in languages other than English due to resource constraints. Males residing in remote and very remote areas were not included and although accounting for less than 3 % of the population, this group is known to have worse health outcomes than other regional groups and includes a larger proportion of indigenous males who also have greater disease burden and premature mortality [2].

## Conclusions

Ten to Men is a unique resource for research into male health both in Australia and more broadly. It has the benefit of a large national sample, a broad age-range, and strong regional representation. The use of common measures across all age groups will facilitate the development of a more comprehensive understanding of male health across the life course and provides the potential to observe multiple key life transitions. Moreover the adoption of measures used in other major Australian and international data collections provides opportunities for comparison and for filling gaps that other studies do not currently address. Increasingly researchers in population health acknowledge the importance of determinants of health at the social and environmental level. The inclusion of a suite of social determinant constructs including income, financial security, housing type & tenure, education, employment, working conditions, social support, community engagement, household structure and gender roles adds an important dimension to the study. Researchers will be able to investigate social/environmental and individual level factors influencing health behaviours and outcomes in males and over the longitudinal course of the study identify causal pathways across those levels. This in turn will support the development of policies and interventions to address the disparity in disease burden and premature mortality in Australian males.
